# Evaluation of in vitro activity of delafloxacin against *Staphylococcus aureus* MSSA and MRSA isolated from a Brazilian tertiary hospital

**DOI:** 10.1007/s42770-026-02084-7

**Published:** 2026-09-14

**Authors:** Patricia Orlandi Barth, Laura Ribas Ucha, Larissa Lutz, Afonso Luís Barth

**Affiliations:** 1 Laboratório de Pesquisa em Resistência Bacteriana, Hospital de Clínicas de Porto Alegre, Porto Alegre, RS Brazil; 2 Programa de Pós-Graduação em Ciências Médicas, Universidade Federal do Rio Grande do Sul, Porto Alegre, RS Brazil; 3 Unidade de Microbiologia e Biologia Molecular, Hospital de Clínicas de Porto Alegre, Porto Alegre, RS Brazil

**Keywords:** *Staphylococcus aureus*, Delafloxacin, Skin and soft tissue infections, MRSA, MSSA, IR Biotyper

## Abstract

**Introduction:**

Proper treatment for infections caused by *Staphylococcus aureus*, particularly methicillin-resistant strains (MRSA), remains a constant challenge in hospital settings. In this context, the search for new antimicrobials, such as delafloxacin, has become necessary. Delafloxacin is a novel quinolone with a broad spectrum of activity against both gram-positive and gram-negative bacteria, offering a new therapeutic option for the treatment of infections caused by multidrug-resistant bacteria.

**Materials and methods:**

117 *S. aureus* isolates were included in this study: 48 *S. aureus* methicillin-susceptible (MSSA) and 69 *S. aureus* methicillin-resistant (MRSA). Isolates were recovered from skin and soft tissue sites, respiratory tract, and blood cultures. Delafloxacin susceptibility was assessed using the gradient diffusion strip (Liofilchem^®^), and the results were interpreted according to the BrCAST guidelines. To assess the clonal profile of *S. aureus*, Fourier-transform infrared (FTIR) spectroscopy was employed using the IR Biotyper system.

**Results:**

Susceptibility to delafloxacin varied from 97.8% to 54.7% for isolates from skin/soft tissue and from respiratory tract, respectively. Blood culture isolates exhibited a Minimum Inhibitory Concentration (MIC) ≤ 0.5 mg/L. FTIR analysis using the IR Biotyper indicated a diverse distribution among the isolates.

**Discussion:**

Due to the high sensitivity rates found in this study, delafloxacin may be considered as a good therapeutic option for the treatment of *S. aureus* infections, including MRSA, particularly in skin and soft tissue infections. The IR Biotyper analysis revealed considerable clonal variation among the isolates, enhancing the robustness of the study.

**Supplementary Information:**

The online version contains supplementary material available at https://doi.org/10.1007/s42770-026-02084-7.

## Introduction

Skin and Soft Tissue Infections (SSTI) encompass a variety of pathological conditions involving the skin and underlying tissues (subcutaneous tissue, fascia, or muscle), ranging from simple superficial infections to severe necrotizing infections. Therefore, they are considered a common clinical issue in surgical departments [1]. The predominant bacterial species associated with SSTI include *Staphylococcus aureus* and other gram-positive organisms such as *Streptococcus pyogenes* and *Enterococcus* spp., as well as gram-negative species like *Pseudomonas aeruginosa* and *Escherichia coli* [2]. *S. aureus* has been commonly documented as one of the most important pathogens among gram-positive cocci, both community-acquired and hospital-associated infections. In particular, methicillin-resistant *S. aureus* (MRSA) is one of the most concerning due to its resistance to all β-lactam antibiotics [3].

The progressive increase in methicillin resistance among *S. aureus* over the past few decades has led to additional therapeutic challenges, leading to high rates of inadequate antibiotic treatment and a significant increase in morbidity, mortality, and healthcare costs [4]. Furthermore, MRSA isolates causing skin infections are often also resistant to macrolides and fluoroquinolones [5, 6]. These concerns have driven efforts to develop new antimicrobial agents that are effective against these pathogens, with some agents approved in the United States in the past decade and others under development for the treatment of SSTI [7].

Among these new therapeutic options, it is of note the delafloxacin, an antimicrobial agent from the quinolone family with broad activity against gram-positive organisms, including MRSA, and gram-negative organisms [8]. Chemically, delafloxacin is a fluoroquinolone with a distinctive chemical structure, characterized by the absence of a strongly basic group at the C-7 position and a heteroaromatic substitution at the N-1 position. These structural features confer a weakly acidic character to the molecule and enhance its antibacterial activity under acidic conditions [9, 10]. It has been recently approved in Brazil for the treatment of SSTI based on its efficacy compared to vancomycin and linezolid, which are considered first-line antimicrobials for treating these infections [8, 11]. In addition to its activity against *S. aureus* in SSTI, delafloxacin is also indicated for the treatment of respiratory infections (community-acquired pneumonia) caused by *S. aureus* [12]. Delafloxacin has shown high microbiological response rates against levofloxacin-non-susceptible isolates in a global study involving patients with SSTI [13].

Therefore, the aim of this study was to evaluate the in vitro activity of delafloxacin against *S. aureus* isolates, both methicillin-susceptible (MSSA) and MRSA. Additionally, the study aimed to assess the clonal relationship of these isolates using molecular typing by Fourier-transform infrared (FTIR) spectroscopy, employing the IR Biotyper system, in order to evaluate the clonality of the isolates.

## Materials and methods

### Selection and identification of isolates

One hundred and seventeen clinical *S. aureus* isolates collected between 2023 and 2024 at Hospital de Clínicas de Porto Alegre were selected from skin and soft tissue materials (*n* = 47), respiratory tract (*n* = 53), and blood cultures (*n* = 17). All isolates were identified by matrix-assisted laser desorption/ionization time-of-flight mass spectrometry (MALDI-TOF–MS), with a Microflex LT^®^, V13.0 database (Bruker Daltonics, Bremen, Germany). Isolates were kept in glycerol broth under − 80 °C before the susceptibility tests.

Isolates were either methicillin-susceptible (*n* = 48) and methicillin-resistant (*n* = 69). Although there is no established breakpoint in the BrCAST guidelines for delafloxacin tested against *S. aureus* from bloodstream infections, blood cultures were selected to assess the minimum inhibitory concentration (MIC) of these isolates. Susceptibility to oxacillin and levofloxacin was also previously assessed by disk diffusion, according to BrCAST guidelines [14].

### Evaluation of delafloxacin susceptibility

The isolates were thawed and subcultured at 35–37 °C on Blood Agar for 18–20 h. A second subculture was made on Mueller-Hinton (MH) Agar. The Minimum Inhibitory Concentration (MIC) of each isolate was determined by gradient strips tests (Liofilchelm^®^). The isolates were classified as susceptible when the MIC was ≤ 0.25 mg/L or ≤ 0.016 mg/L for SSTI and respiratory infections, respectively. Isolates from blood cultures were not interpreted as susceptible or resistant due to the lack of breakpoints by BrCAST of Delafloxacin for *S.aureus.*

### IR Biotyper^®^

A subset of 58 isolates were randomly selected for evaluation of clonal relatedness with FTIR using IR Biotyper equipment. The isolates were subcultured as described above. For the preparation of the technique, two loopful of bacterial culture (2 µL) growth in MH were resuspended in 50 µL of 70% ethanol in a 1.5 mL Eppendorf tube containing sterile metal rods. To achieve a uniform suspension, vortexing was performed for 1 min. Afterwards, 50 µL of sterile water was added, and the final suspension of 100 µL volume was vortexed for an additional minute. Subsequently, 15 µL of the bacterial suspension was applied in triplicate on the IR Biotyper plate and allowed to dry at room temperature. Internal controls (IRTS1) and (IRTS2) from the IR Biotyper kit were used as quality assurance for the spots prepared. The isolates were phenotypically characterized using FTIR methodology according to the manufacturer’s instructions. Spectra were acquired using OPUS 7.5 software (Bruker Daltonics GmbH & Co. KG, Bremen, Germany). The spectrum range of 1,300–800 cm^− 1^ was automatically processed and normalized, and second derivatives were applied by the IR Biotyper software. The data were analyzed with IR Biotyper software by verifying the distribution of the isolates with principal component analysis (PCA) for dimensionality reduction into 2D graphic.

## Results

Among the 100 clinical isolates from SSTIs and respiratory tract infections evaluated for delafloxacin susceptibility, 75 (75.0%) were susceptible and 25 (25.0%) were resistant. Delafloxacin showed markedly higher activity against SSTI isolates, with 46 of 47 isolates (97.9%) classified as susceptible and only one isolate (2.1%) classified as resistant. In contrast, among the 53 respiratory tract isolates, 29 (54.7%) were susceptible and 24 (45.3%) were resistant. When stratified according to methicillin susceptibility, delafloxacin showed similar overall susceptibility rates among MSSA and MRSA isolates (74.4% and 75.4%, respectively). However, differences were observed according to the infection site: among SSTI isolates, susceptibility was 100% for MSSA and 97.0% for MRSA, whereas among respiratory isolates, susceptibility was 62.1% for MSSA and 45.8% for MRSA (Table 1). All seventeen blood culture isolates presented a Minimum Inhibitory Concentration (MIC) ≤ 0.5 mg/L.

**Table 1:** Delafloxacin susceptibility among *Staphylococcus aureus* isolates according to infection site and methicillin susceptibility

Sample type	Methicillin phenotype	*n*	Susceptible, *n* (%)	Resistant, *n* (%)
SST	MSSA	14	14 (100.0)	0 (0.0)
SST	MRSA	33	32 (97.0)	1 (3.0)
SST – total		47	46 (97.9)	1 (2.1)
Respiratory tract	MSSA	29	18 (62.1)	11 (37.9)
Respiratory tract	MRSA	24	11 (45.8)	13 (54.2)
Respiratory tract – total		53	29 (54.7)	24 (45.3)
Total	MSSA	43	32 (74.4)	11 (25.6)
Total	MRSA	57	43 (75.4)	14 (24.6)
Overall		100	75 (75.0)	25 (25.0)

Considering all isolates, the MIC_50_ and MIC_90_ values were 0.008 mg/L and 0.25 mg/L, respectively (Supplementary Material Table S1).

**Fig. 1:**
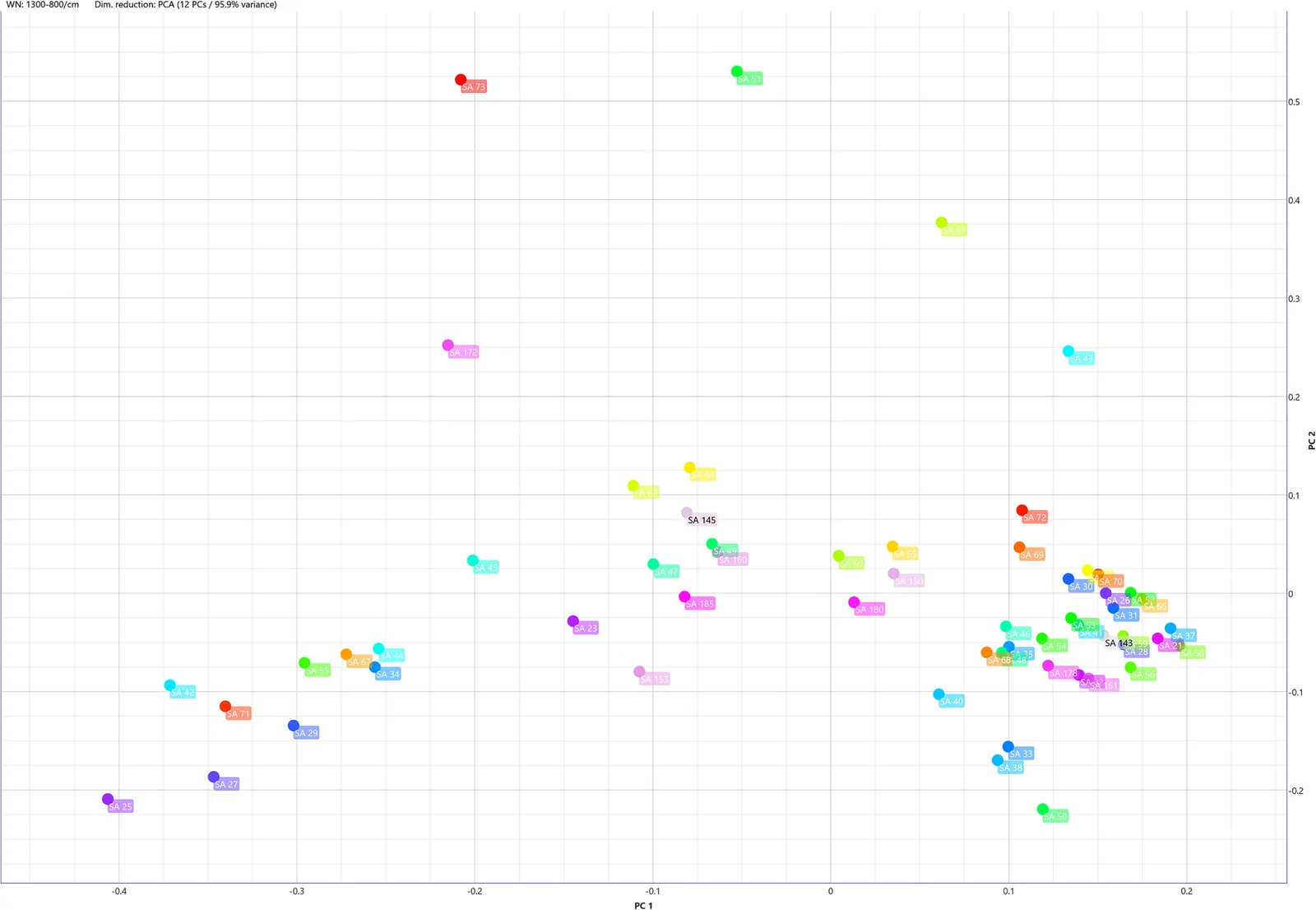
Two-dimensional principal component analysis (PCA) of the *Staphylococcus aureus* isolates based on Fourier-transform infrared (FTIR) spectral profiles. Each point represents one isolate. The relative position of the points reflects the similarity of their FTIR spectral profiles, with isolates located closer together showing greater spectral similarity. The absence of a clearly defined cluster indicates a heterogeneous distribution of the isolates, without evidence of a predominant group with highly similar spectral profiles

The evaluation of the relatedness of the isolates by FTIR showed a heterogeneous distribution in the two-dimensional PCA, without a clearly defined predominant cluster. Isolates located closer to each other in the PCA space exhibit greater spectral similarity, whereas the absence of a well-defined cluster suggests that the isolates do not comprise a predominant group with a highly similar spectral profile [15] (Fig. 1).

## Discussion

New antimicrobial options are essential to reduce morbidity, mortality, and healthcare-related costs due to infections associated with MRSA [16]. Delafloxacin is a fluoroquinolone with broad-spectrum activity against Gram-positive pathogens, including MRSA as well as Gram-negative pathogens [17]. Delafloxacin has been approved for clinical use in several countries, including the United States, where it is indicated for acute bacterial skin and skin structure infections and community-acquired bacterial pneumonia, and in the European Union, where it is approved for the same indications, although its use for community-acquired pneumonia is recommended when other commonly recommended antibacterial agents are considered inappropriate [18, 19]. Regarding in vitro susceptibility profiles, prior studies indicated that delafloxacin in comparison with other quinolones presented increased susceptibility rates [20–22]. In Brazil, delafloxacin is also registered for the treatment of severe skin and soft tissue infections and community-acquired bacterial pneumonia [11]. Despite its regulatory approval in the country, robust data describing its current utilization are limited.

In the present study, delafloxacin demonstrated significant in vitro activity against *S. aureus* isolated from SSTIs, regardless of methicillin susceptibility. Therefore, this fluoroquinolone can be considered as an effective therapeutic option for the treatment of these infections, since among all isolates recovered from skin and soft tissue, only a single isolate was resistant to delafloxacin in vitro. For community-acquired pneumonia, delafloxacin may also be considered as a treatment option, although it was not as effective as in vitro as observed for SSTIs. It is important to note that the MIC susceptibility breakpoint for community-acquired pneumonia (≤ 0.016 mg/L) is considerably lower than that established for SSTIs (≤ 0.25 mg/L). Consequently, isolates with MICs > 0.016 to 0.25 mg/L are classified as resistant when interpreted according to the breakpoint for community-acquired pneumonia but as susceptible when the SSTI breakpoint is applied. This difference in interpretive criteria may have contributed to the higher resistance rate observed among respiratory isolates.

All isolates evaluated in this study had previously been tested for susceptibility to levofloxacin, a quinolone belonging to the same class as delafloxacin. Our results showed that among levofloxacin-resistant isolates (*n* = 46), delafloxacin retained in vitro inhibitory activity in a substantial proportion (*n* = 25; 54.3%), indicating that the mechanisms conferring resistance to levofloxacin do not necessarily result in resistance to delafloxacin. This finding can be explained by the unique chemical structure of delafloxacin, which differentiates it from other quinolones and includes: (1) the presence of a substituted heteroaromatic ring that enhances bactericidal activity; (2) lower polarity, which increases its potency against Gram-positive organisms resistant to traditional quinolones; and (3) the absence of a basic group, rendering the molecule weakly acidic and increasing its activity in acidic environments, such as those found in skin and soft tissue infections [23].

This study evaluated the effectiveness of delafloxacin against both MSSA and MRSA isolates and found similar susceptibility rates of 74.4% and 75.4%, respectively, demonstrating the considerable therapeutic potential of this antimicrobial, particularly in settings where other treatment options may be limited. These results are in agreement with findings from a previous Brazilian study; however, the earlier investigation evaluated a smaller sample size from different Brazilian regions [24].

The analysis of the relatedness of the evaluated isolates using the IR Biotyper showed a heterogeneous distribution, with no clearly defined predominant cluster. This finding suggests that the isolates represented diverse spectral profiles rather than a single predominant clonal group. This heterogeneity is particularly relevant to the interpretation of the delafloxacin susceptibility profile, as it supports that the observed susceptibility pattern reflects the activity of delafloxacin across genetically diverse isolates rather than being driven predominantly by a single disseminated clone.

The results of this study demonstrated significant in vitro activity of delafloxacin against *S. aureus*, particularly for isolates causing SSTIs, indicating that this novel quinolone may represent a promising therapeutic option for the treatment of skin and soft tissue infections and community-acquired pneumonia caused by *Staphylococcus aureus*, including both meticilin-resistant and meticilin-susceptible isolates.

## Supplementary Information

Below is the link to the electronic supplementary material.

Supplementary Material 1 (DOCX 685 KB)

## Data Availability

All data relevant to the findings of this study are included in the article. Additional information or data may be made available by the corresponding author upon reasonable request.
