# Supplementary material for: Evaluation of in vitro activity of delafloxacin against Staphylococcus aureus MSSA and MRSA isolated from a Brazilian tertiary hospital
Source: Braz J Microbiol. 2026 Sep 14;57(1):270. doi: 10.1007/s42770-026-02084-7 (PMC13575000; doi:10.1007/s42770-026-02084-7)
Supplement: Supplementary file 1 [file 42770_2026_2084_MOESM1_ESM.docx]

**Table S1:** **Individual susceptibility profiles of the *Staphylococcus aureus* isolates included in the study.**

| **Isolate** |  | **Sample** | **Levofloxacin** | **Oxacilin** | **Delafloxacin**  **(mg/L)** | **Delafloxacin**  **Interpretation** |
| --- | --- | --- | --- | --- | --- | --- |
| 173 |  | SST | R | R | 0.190 | S |
| 174 |  | Blood | I | R | <0.002 | NI |
| 175 |  | SST | I | S | 0.004 | S |
| 177 |  | RT | R | S | 0.125 | R |
| 178 |  | RT | R | S | 0.064 | R |
| 179 |  | RT | R | S | 0.094 | R |
| 180 |  | RT | I | S | <0.002 | S |
| 181 |  | RT | I | S | 0.064 | R |
| 182 |  | RT | I | S | 0.064 | R |
| 183 |  | RT | I | S | <0.002 | S |
| 184 |  | Blood | I | S | 0.004 | NI |
| 185 |  | SST | I | S | <0.002 | S |
| 186 |  | RT | I | S | <0.002 | S |
| 187 |  | RT | I | S | 0.002 | S |
| 188 |  | RT | I | S | 0.003 | S |
| 189 |  | Blood | I | S | <0.002 | NI |
| 190 |  | Blood | I | S | 0.004 | NI |
| 191 |  | RT | I | S | 0.004 | S |
| 192 |  | RT | I | S | 0.003 | S |
| 193 |  | SST | I | S | 0.064 | S |
| 194 |  | RT | I | S | <0.002 | S |
| 195 |  | RT | I | S | 0.064 | R |
| 196 |  | SST | I | S | 0.004 | S |
| 197 |  | RT | R | S | 0.125 | R |
| 198 |  | SST | I | S | 0.004 | S |
| 199 |  | RT | R | R | 0.190 | R |
| 200 |  | SST | R | R | 0.125 | S |
| 201 |  | SST | I | S | <0.002 | S |
| 202 |  | RT | I | S | 0.064 | R |
| 204 |  | RT | I | S | <0.002 | S |
| 205 |  | RT | I | S | 0.003 | S |
| 206 |  | SST | I | S | 0.003 | S |
| 207 |  | SST | I | S | <0.002 | S |
| 208 |  | Blood | I | S | <0.002 | NI |
| 209 |  | RT | I | S | <0.002 | S |
| 210 |  | SST | I | S | <0.002 | S |
| 212 |  | RT | R | S | 0.250 | R |
| E4 |  | RT | I | S | <0.002 | S |
| E5 |  | Blood | I | S | 0.004 | NI |
| E6 |  | RT | I | S | 0.004 | S |
| E7 |  | RT | I | S | <0.002 | S |
| E8 |  | RT | I | S | <0.002 | S |
| S.A.1 |  | SST | I | R | 0.003 | S |
| S.A.2 |  | RT | I | R | 0.003 | S |
| S.A.3 |  | RT | R | R | 0.094 | R |
| S.A.4 |  | RT | I | R | 0.006 | S |
| S.A.6 |  | SST | R | R | 0.125 | S |
| S.A.7 |  | RT | I | R | 0.004 | S |
| S.A.8 |  | SST | I | R | 0.003 | S |
| S.A.9 |  | SST | R | R | 0.125 | S |
| S.A.11 |  | RT | R | R | 0.094 | R |
| S.A.12 |  | RT | R | R | 0.047 | R |
| S.A.13 |  | SST | R | R | 0.190 | S |
| S.A.14 |  | SST | R | R | 0.190 | S |
| S.A.15 |  | SST | R | R | 0.125 | S |
| S.A.16 |  | SST | I | R | 0.003 | S |
| S.A.18 |  | SST | R | R | 0.125 | S |
| S.A.19 |  | RT | R | R | 0.094 | R |
| S.A. 20 |  | SST | R | R | 0.094 | S |
| S.A. 21 |  | SST | R | R | 0.250 | S |
| S.A. 22 |  | RT | R | R | 0.250 | R |
| S.A.23 |  | RT | I | R | 0.006 | S |
| S.A.25 |  | Blood | I | R | 0.008 | NI |
| S.A.26 |  | Blood | R | R | 0.250 | NI |
| S.A.27 |  | Blood | I | R | 0.008 | NI |
| S.A.28 |  | RT | R | R | 0.500 | R |
| S.A.29 |  | SST | I | R | 0.012 | S |
| S.A.30 |  | SST | R | R | 0.250 | S |
| S.A.31 |  | SST | R | R | 0.190 | S |
| S.A.33 |  | Blood | R | R | 0.380 | NI |
| S.A.34 |  | Blood | I | R | 0.125 | NI |
| S.A.35 |  | SST | R | R | 0.250 | S |
| S.A.37 |  | Blood | I | R | 0.008 | NI |
| S.A.38 |  | SST | R | R | 0.250 | S |
| S.A.40 |  | SST | R | R | 0.250 | S |
| S.A.41 |  | SST | R | R | 0.250 | S |
| S.A.42 |  | RT | I | R | 0.008 | S |
| S.A.43 |  | SST | R | R | 0.250 | S |
| S.A.44 |  | SST | I | R | 0.008 | S |
| S.A.45 |  | SST | I | R | 0.006 | S |
| S.A.46 |  | SST | R | R | 0.190 | S |
| S.A.47 |  | Blood | I | R | <0,002 | NI |
| S.A.48 |  | Blood | R | R | 0.008 | NI |
| S.A.49 |  | RT | I | R | 0.006 | S |
| S.A.50 |  | SST | R | R | 0.125 | S |
| S.A.51 |  | RT | I | R | 0.006 | S |
| S.A.52 |  | SST | R | R | 4.000 | R |
| S.A.53 |  | SST | R | R | 0.125 | S |
| S.A.54 |  | Blood | R | R | 0.380 | NI |
| S.A.55 |  | SST | I | R | 0.008 | S |
| S.A.56 |  | RT | R | R | 0.380 | R |
| S.A.58 |  | RT | R | R | 0.190 | R |
| S.A.59 |  | RT | R | R | 0.380 | R |
| S.A.60 |  | SST | R | R | 0.250 | S |
| S.A.61 |  | Blood | R | R | 0.500 | NI |
| S.A.62 |  | SST | I | R | 0.012 | S |
| S.A.63 |  | RT | R | R | 0.500 | R |
| S.A.64 |  | Blood | I | R | 0.001 | NI |
| S.A.65 |  | RT | R | R | 0.001 | S |
| S.A. 66 |  | RT | R | R | 0.125 | R |
| S.A.67 |  | RT | I | R | 0.004 | S |
| S.A.68 |  | RT | R | R | 0.003 | S |
| S.A.69 |  | RT | R | R | 0.094 | S |
| S.A.70 |  | RT | R | R | 0.190 | R |
| S.A.71 |  | SST | I | R | 0.003 | S |
| S.A. 72 |  | SST | R | R | 0.094 | S |
| S.A.73 |  | SST | I | R | 0.003 | S |
| S.A. 74 |  | RT | R | S | 0.190 | R |
| S.A. 75 |  | RT | I | S | 0.003 | S |
| S.A. 76 |  | SST | I | S | 0.002 | S |
| S.A. 77 |  | SST | I | S | 0.094 | S |
| S.A. 78 |  | RT | I | S | 0.004 | S |
| S.A. 79 |  | RT | R | S | 0.125 | R |
| S.A. 80 |  | SST | I | S | 0.094 | S |
| S.A. 81 |  | RT | I | S | 0.006 | S |
| S.A. 82 |  | SST | I | S | 0.004 | S |
| S.A. 83 |  | SST | I | S | 0.004 | S |

SST = Skin and Soft Tissue; RT = Respiratory Tract; S = Susceptible; I = Susceptible increasing exposure; R = Resistant, NI = Not Interpretable.
